# Construction of a multicontrol sterility system for a maize male‐sterile line and hybrid seed production based on the *ZmMs7* gene encoding a PHD‐finger transcription factor

**DOI:** 10.1111/pbi.12786

**Published:** 2017-08-23

**Authors:** Danfeng Zhang, Suowei Wu, Xueli An, Ke Xie, Zhenying Dong, Yan Zhou, Liwen Xu, Wen Fang, Shensi Liu, Shuangshuang Liu, Taotao Zhu, Jinping Li, Liqun Rao, Jiuran Zhao, Xiangyuan Wan

**Affiliations:** ^1^ College of Bioscience and Biotechnology Hunan Agricultural University Changsha China; ^2^ Advanced Biotechnology and Application Research Center School of Chemistry and Biological Engineering University of Science and Technology Beijing Beijing China; ^3^ Beijing Key Laboratory of Maize DNA Fingerprinting and Molecular Breeding Maize Research Center Beijing Academy of Agriculture & Forestry Sciences Beijing China; ^4^ Beijing Engineering Laboratory of Main Crop Biotechnology Breeding Beijing Solidwill Sci‐Tech Co. Ltd. Beijing China

**Keywords:** *ZmMs7*, genetic male sterility, hybrid seed production, transgenic maintainer line, nontransgenic progeny, maize

## Abstract

Although hundreds of genetic male sterility (GMS) mutants have been identified in maize, few are commercially used due to a lack of effective methods to produce large quantities of pure male‐sterile seeds. Here, we develop a multicontrol sterility (MCS) system based on the maize *male sterility 7* (*ms7*) mutant and its wild‐type *Zea mays Male sterility 7* (*ZmMs7*) gene via a transgenic strategy, leading to the utilization of GMS in hybrid seed production. *ZmMs7* is isolated by a map‐based cloning approach and encodes a PHD‐finger transcription factor orthologous to rice PTC1 and *Arabidopsis* MS1. The MCS transgenic maintainer lines are developed based on the *ms7‐6007* mutant transformed with MCS constructs containing the (i) *ZmMs7* gene to restore fertility, (ii) α‐amylase gene *ZmAA* and/or (iii) DNA adenine methylase gene *Dam* to devitalize transgenic pollen, (iv) red fluorescence protein gene *DsRed2* or *mCherry* to mark transgenic seeds and (v) herbicide‐resistant gene *Bar* for transgenic seed selection. Self‐pollination of the MCS transgenic maintainer line produces transgenic red fluorescent seeds and nontransgenic normal colour seeds at a 1:1 ratio. Among them, all the fluorescent seeds are male fertile, but the seeds with a normal colour are male sterile. Cross‐pollination of the transgenic plants to male‐sterile plants propagates male‐sterile seeds with high purity. Moreover, the transgene transmission rate through pollen of transgenic plants harbouring two pollen‐disrupted genes is lower than that containing one pollen‐disrupted gene. The MCS system has great potential to enhance the efficiency of maize male‐sterile line propagation and commercial hybrid seed production.

## Introduction

Maize is one of the most important crops that have been successfully applied to achieve heterosis. Heterosis is a phenomenon in which heterozygous hybrid progeny are superior to both homozygous parents. To produce pure hybrid seeds, the female inbred parent line must be prevented from undergoing self‐pollination and should be directionally crossed to the male inbred parent because maize is a monoecious plant. During maize hybrid seed production, many methods can be used to prevent self‐pollination of the female parent line, such as manual or mechanical emasculation (detasseling), application of male‐specific gametocides and use of cytoplasmic or nuclear‐encoded male sterility (Perez‐Prat and van Lookeren Campagne, 2002).

Manual or mechanical detasseling, namely the physical removal of the male floral structure, remains the predominant method in commercial hybrid seed production in maize. However, this technique is not only time‐consuming, labour‐intensive and expensive but also detrimental to plant growth, thus reducing the yield of hybrid seeds because a portion of the plant photosynthetic body is destroyed during emasculation (Skibbe and Schnable, 2005; Wych, 1988). Although a number of chemicals have been investigated as potential gametocides for foliar sprays prior to flowering in commercial maize seed production (McRae, 1985), there has been little industrial use of chemical sterilants because of the risk of incomplete pollen sterility, a reduction in female fertility and its detrimental effect on the environment.

Male sterility can also be generated by either cytoplasmic or nuclear genes. Cytoplasmic male sterility (CMS) has been used in commercial hybrid maize production, but the CMS system suffers from several intrinsic problems, including the poor genetic diversity between the CMS lines and the restoration lines, increased disease susceptibility, a breakdown of sterility in certain environments or genetic backgrounds, and unreliable restoration (Williams, 1995). Nuclear‐encoded male sterility (NMS) or GMS results from mutations in the nuclear genome, which is a common spontaneous phenomenon in flowering plants (Kaul, 1988). To date, hundreds of genetic male‐sterile mutants have been identified and described in maize (Timofejeva *et al*., 2013). Most of these mutants are controlled by a recessive gene, which provides an excellent genetic means of emasculation for hybrid seed production in maize. However, it is not possible to obtain a pure increase in male‐sterile homozygous female inbred seeds through traditional self‐pollination because the plant is male sterile, and the largest percentage of male‐sterile seeds that can be produced is 50% (Williams, 1995). Fortunately, the rapid development of gene cloning, recombinant DNA and plant transformation techniques have provided new possibilities for creating genetically engineered male sterility.

Since the first transgenic male sterility system was described (Mariani *et al*., 1990), many strategies to produce male‐sterile plants have been reported,including chemical inducible male sterility systems (Feng *et al*., 2014; Singh *et al*., 2010; Venkatesh *et al*., 2014), transgenic maintainer systems (Chang *et al*., 2016; Fox *et al*., 2017; Perez‐Prat and van Lookeren Campagne, 2002; Williams, 1995; Wu *et al*., 2016) and other biotechnology‐based systems (Gils *et al*., 2008; Millwood *et al*., 2016; Mitsuda *et al*., 2006). Among them, the strategy of using transgenic maintainer lines to propagate nuclear male‐sterile plants is more desirable and reliable. The transgenic maintainer lines are obtained by transforming dominant or recessive male‐sterile plants with a construct comprising three modules: a male sterility complementary fertility restoration gene, a pollen‐lethality gene to disrupt the transgenic pollen and a seed colour marker gene for mechanical colour sorting of the seeds (Perez‐Prat and van Lookeren Campagne, 2002). Self‐pollination of the resulting maintainer lines propagates 50% of maintainer seeds and 50% of male‐sterile seeds, which can be sorted based on the seed colour marker. Cross‐pollination of the maintainer line to the male‐sterile line produces 100% male‐sterile seeds. To successfully apply the maintainer line, the basic prerequisite of this strategy is to identify and clone the male sterility gene, which is usually obtained from a male‐sterile mutant.

Despite the large collection of male‐sterile mutants in maize, only a handful of the mutants have been characterized cytologically, and even fewer genes have been cloned and studied in detail, such as *ms8* (Wang *et al*., 2010, 2012b, 2013), *ms9* (Albertsen *et al*., 2016), *ms22/msca1* (Albertsen *et al*., 2009; Chaubal *et al*., 2003), *ms23* (Nan *et al*., 2017), *ms26* (Djukanovic *et al*., 2013), *ms32* (Moon *et al*., 2013), *Ms44* (Fox *et al*., 2017), *ms45* (Cigan *et al*., 2001), *apv1* (Somaratne *et al*., 2017), *ipe1* (Chen *et al*., 2017), *mac1* (Wang *et al*., 2012a) and *ocl4* (Vernoud *et al*., 2009). The cloning and functional characterization of these male sterility genes have contributed significantly to our understanding of the molecular mechanism underlying anther development in maize and provided useful gene resources for genetic engineering of male‐sterile lines in hybrid seed production. However, compared with the related studies in rice and *Arabidopsis*, the molecular mechanism responsible for most maize male‐sterile mutants is largely unknown. Based on the phenotypic and cytological similarity of male‐sterile mutants among maize, rice and *Arabidopsis*, the cloned male sterility genes in rice and *Arabidopsis* can be used for reference in maize. For example, the *Arabidopsis MALE STERILITY1* (*AtMS1*) gene encodes a PHD‐finger transcription factor and regulates pollen and tapetum development. *AtMS1* is expressed specifically in the tapetum after meiosis and is involved in formation of the pollen exine and pollen cytosolic components as well as development of the tapetum (Ito and Shinozaki, 2002; Ito *et al*., 2007; Wilson *et al*., 2001; Yang *et al*., 2007). In rice, *PERSISTENT TAPETAL CELL1* (*OsPTC1*), the ortholog of *AtMS1*, controls programmed tapetal cell death and functional pollen development. Additionally, the rice *ptc1* mutant is phenotypically similar to the *Arabidopsis ms1* mutant in terms of a lack of tapetal DNA fragmentation, delayed tapetal degeneration, abnormal pollen wall formation and aborted microspore development. The *ptc1* mutant displays uniquely uncontrolled tapetal proliferation and subsequent commencement of necrosis‐like tapetal death (Li *et al*., 2011). However, to date, the ortholog of *Arabidopsis* MS1 and rice PTC1 in maize remains undescribed.

Based on the critical time points of male sterility conversion, the male‐sterile mutants can be classified as premeiotic, meiotic and postmeiotic. In previous reports, hundreds of maize male‐sterile mutants were identified and classified into the above three classes based on cytological characterization and allelism testing (Timofejeva *et al*., 2013). Twenty‐two maize male‐sterile mutants with premeiotic developmental defects were used to evaluate the detailed cytological characteristics. Four general types of premeiotic defects were classified: anther identity defects, abnormal anther structure, anther wall layer defects and premature cell layer degradation (Timofejeva *et al*., 2013). Among the male‐sterile genes mentioned above, *ms8*,* ms22/msca1*,* ms23*,* ms32*,* mac1* and *ocl4* were premeiotic mutants, while *ms26*,* ms45*,* apv1* and *ipe1* were postmeiotic mutants. Moreover, among the premeiotic mutants, *ms8* was classified into the group of premature cell layer degradation, *ms22/msca1* was classified into the group of anther identity defects, and *ms23*,* ms32*,* mac1* and *ocl4* were classified into the group of anther wall layer defects (Timofejeva *et al*., 2013). Furthermore, maize *ms7* was a historic male‐sterile mutant and had been previously studied morphologically and cytologically (Albertsen and Phillips, 1981; Beadle, 1932; Morton *et al*., 1989). In 1981, the *ms7* mutant was described as having a poorly developed, thin pollen wall and premature vacuolization and degradation of tapetal cells (Albertsen and Phillips, 1981). Morton *et al*. (1989) confirmed that the *ms7* mutant had a relatively thin microspore wall, a poorly developed microspore aperture and several tapetal cell and orbicule morphological abnormalities. However, no visible defects were observed in *ms7* anthers prior to tetrad formation (Morton *et al*., 1989). Therefore, the *ms7* mutant should be classified as a postmeiotic developmental mutant with abnormal tapetal cell layer degeneration, in contrast to the above‐described mutants based on cytological observation. Nevertheless, the mutated *ms7* gene and its role in anther development are largely unknown.

In this study, the maize wild‐type *ZmMs7* gene was isolated by a map‐based cloning approach. *ZmMs7* encodes a PHD‐finger transcription factor that is orthologous to rice PTC1 and *Arabidopsis* MS1*. ZmMs7* was characterized with respect to spatio‐temporal expression patterns, cytological and molecular biological analyses and transgenic complementation. Based on the *ZmMs7* gene, a MCS system was developed and preliminarily evaluated. This work will provide new insights into the molecular mechanism of male sterility in maize and may enhance the efficiency of maize male‐sterile line propagation and hybrid seed production.

## Results

### *ms7* is a single recessive mutant that exhibits complete male sterility in maize

**Figure 1:**
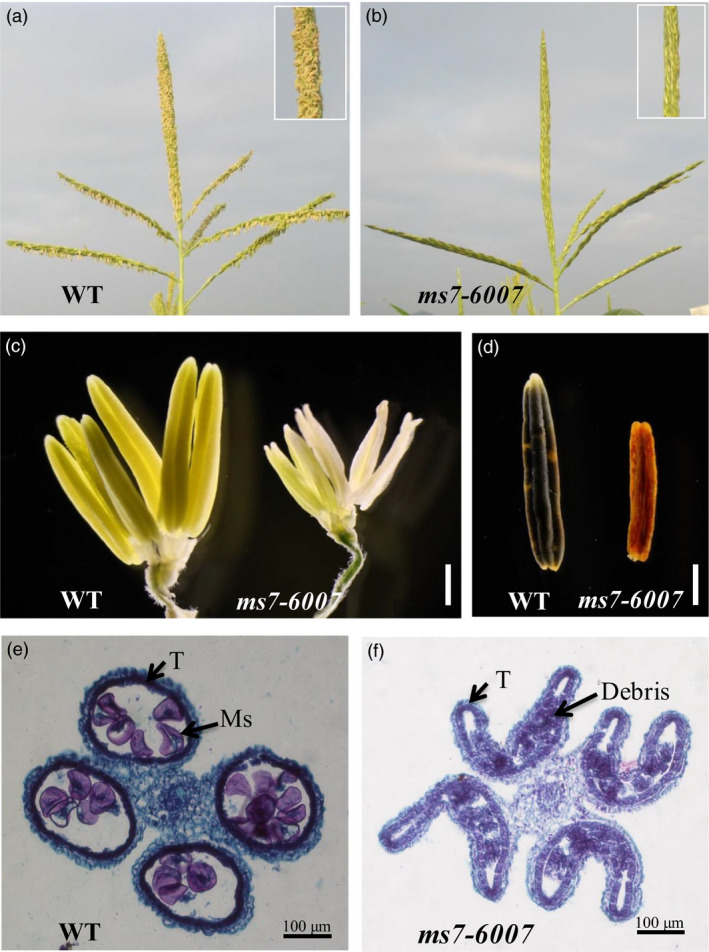
Phenotypic comparison of the wild type (WT) and *ms7‐6007* mutant. (a and b) The tassel of WT (a) and *ms7‐6007* (b). (c) The spikelet of WT (left) and *ms7‐6007* (right) with the glume, lemma and palea removed. (d) The anther of WT (left) and *ms7‐6007* (right) stained with I_2_‐KI. (e and f) Transverse sections of the entire anther of wild type (e) and *ms7‐6007* (f) in the late microspore developmental stage. T, tapetum; Ms, microspore. Bars = 1 mm (c, d), 100 μm (E, F).

Two alleles of *ms7* mutant, *ms7‐6007* and *ms7gl1*, were obtained from the Maize Genetics Cooperation Stock Center (http://maizecoop.cropsci.uiuc.edu). Compared with the wild‐type male‐fertile sibling, *ms7‐6007* displayed complete male sterility with no exserted anthers (Figure 1a and b) but normal vegetative growth and female fertility. The mutant anthers were small and whitish (Figure 1c), lacking pollen grains, and were resistant to I_2_‐KI staining (Figure 1d). Additionally, the mutant phenotype was stable in multiple environments including different years and locations. The *ms7gl1* mutant also exhibited a similar male‐sterile phenotype to the *ms7‐6007* mutant (Figure S1).

To investigate the cytological defects of the *ms7* mutant in anther development, a microscopic comparison analysis between *ms7‐6007* and wild type was conducted. During the late microspore developmental stage, the wild‐type tapetal cells became condensed and deeply stained, and they gradually degenerated via programmed cell death (PCD, Figure 1e). In contrast, the *ms7‐6007* mutant tapetal cells were swollen and lightly stained, and the microspore was aborted, leaving debris in the shrinkage locule (Figure 1f) indicative of abnormal tapetal cell PCD.

When the two *ms7* mutants were crossed with the normal maize inbred line Chang7‐2, all of the F_1_ progeny was male fertile and the F_2_ population displayed a 3:1 segregation of male fertile to sterile plants (Table S1), suggesting that both *ms7‐6007* and *ms7gl1* were single recessive mutations. Furthermore, when the *ms7‐6007/ms7‐6007* homozygous plants were outcrossed using pollen from *ms7gl1/+* heterozygous plants, the F_1_ progeny showed a 1:1 segregation ratio of male sterile to fertile plants, implying that the mutant gene of *ms7‐6007* is allelic to that of *ms7gl1*.

### Isolation of the *ZmMs7* male fertility gene

**Figure 2:**
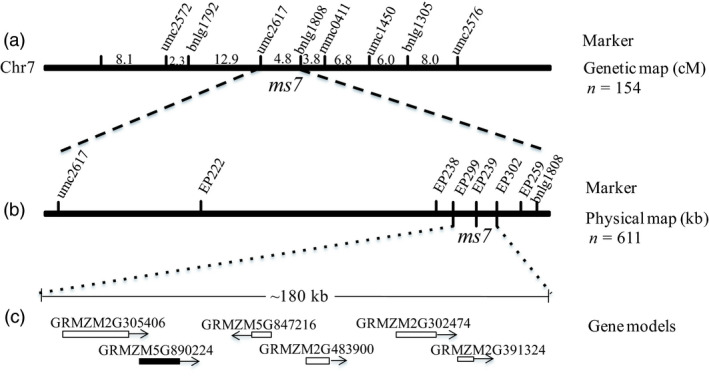
Map‐based cloning of the maize *ms7* gene. (a) primary mapping of the *ms7* gene on maize chromosome 7 between SSR markers umc2617 and bnlg1808. (b) Fine mapping of the *ms7* gene to an interval of nearly 180 kb between CAPS markers EP299 and EP302. (c) The six putative gene models in the interval. Among them, *GRMZM5G890224*, similar to the rice *PTC1* gene, is the candidate gene. n, The number of the male‐sterile plants used in the F_2_ mapping population.

The *ms7* mutant gene was identified by the map‐based cloning approach. Using a F_2_ population that included 154 male‐sterile individuals, the *ms7* locus was initially mapped to chromosome 7 between SSR markers umc2617 and bnlg1808 with a genetic distance of 4.8 cM (Figure 2a). Then, based on the maize genome sequence information, six CAPS markers in the interval were designed for fine mapping of the *ms7* gene in an enlarged F_2_ population that included 611 male‐sterile individuals. Finally, the *ms7* gene was defined to the interval with a 180‐kb length between EP299 and EP302 (Figure 2b). Six predicted genes are located in this region (Figure 2c), including *GRMZM5G890224*, which encodes a putative PHD‐finger protein.

**Figure 3:**
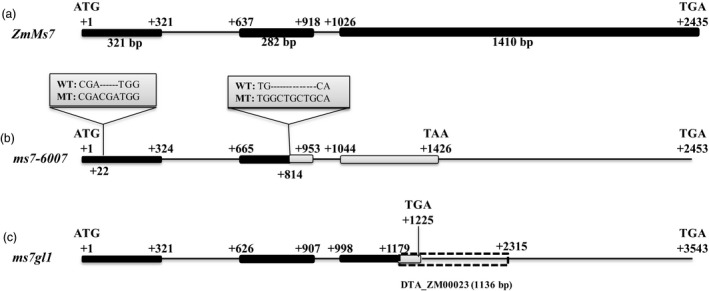
The gene structure of the maize *ZmMs7* candidate gene (*GRMZM5G890224*). A schematic representation of three exons and two introns of *ZmMs7* in wild type (a) *ms7‐6007* (b) and *ms7gl1* (c). The +1 indicates the putative starting nucleotide of translation, and the stop codon (TGA) is +2435 in wild type. Black boxes indicate exons, and intervening lines indicate introns. Grey boxes indicate the mutant amino acid sequences. Numbers indicate the exon length (bp); the insertion sites in *ms7‐6007* and *ms7gl1* are shown, causing a frameshift mutation and premature stop codon, respectively.

The candidate gene (*GRMZM5G890224*) consists of three exons and two introns (Figure 3a) and encodes a 670‐amino acid protein. Sequencing of this gene in the *ms7‐6007* mutant revealed a 3‐bp (CGA) insertion at the +22 nucleotide site in the first exon and a 7‐bp (GCTGCTG) insertion at the +814 nucleotide site in the second exon, respectively (Figure 3b). The latter insertion causes a frameshift mutation and alters the reading frame after amino acid residue 157, resulting in a lack of the leucine zipper region and PHD domain (Figure S2). Additionally, sequencing of the target gene in the *ms7gl1* mutant showed that an 1136‐bp transposable element (DTA_ZM00023) is inserted at the +1179 nucleotide site in the third exon (Figure 3c), which also causes an in‐frame premature stop codon and leads to the lack of the leucine zipper region and PHD domain (Figure S2). These results suggested that *GRMZM5G890224* is responsible for the male‐sterile phenotype in the *ms7* mutant, and therefore, *GRMZM5G890224* was tentatively designated *ZmMs7*.

**Figure 4:**
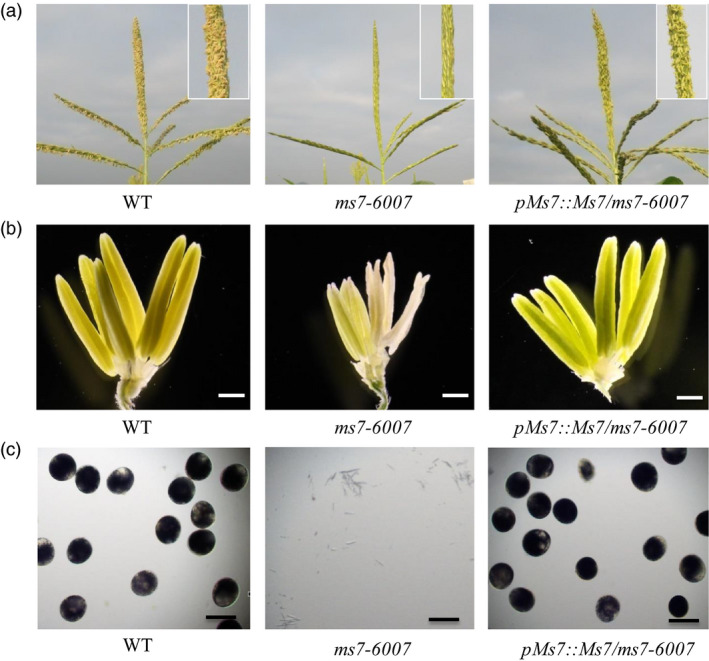
Functional complementation of the maize *ms7‐6007* mutant. Phenotypes of tassels (a), spikelets (b) and pollen grains stained with I_2_‐KI (c) in the wild type, *ms7‐6007* mutant and complemented transgenic line (*pMs7::Ms7/ms7‐6007*). Bars = 1 mm (B), 100 μm (c).

To confirm the above prediction, the functional complementation experiment was performed. The maize Hi‐II hybrid line was transformed with *Agrobacterium tumefaciens* EHA105 containing a *pZmMs7pro::ZmMs7* construct. The transgenic plants were then crossed with the *ms7‐6007* mutant, and transgenic plants in the *ms7‐6007* homozygous mutant background were gained by self‐pollination and marker‐assisted selection. The transgenic plants rescued the male‐sterile defect of *ms7‐6007* and recovered the fertility phenotype (Figure 4), demonstrating that the male‐sterile phenotype of the *ms7‐6007* mutant results from the *ZmMs7* gene mutation.

### *ZmMs7* is an anther‐specific gene

**Figure 5:**
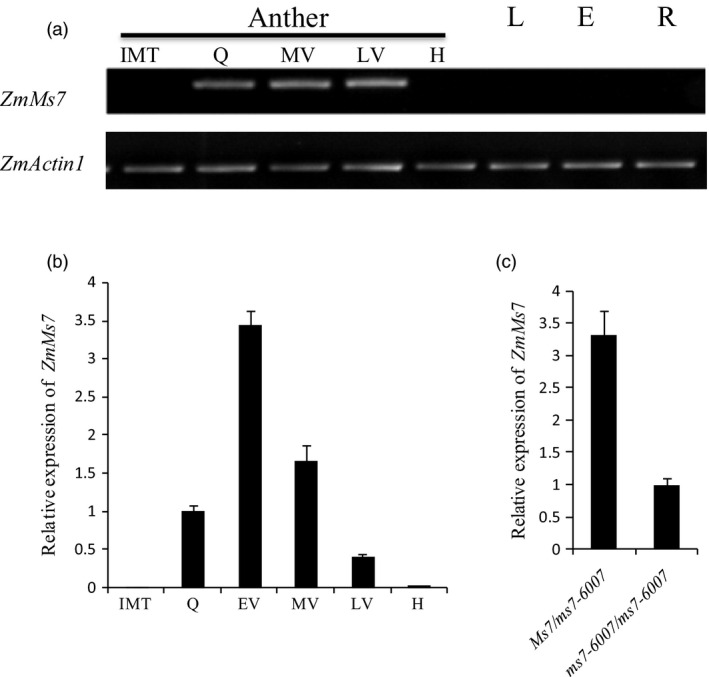
Expression pattern of maize *ZmMs7*. (a) Maximum expression was observed by RT–PCR in the anther during the meiosis quartet to late vacuolate microspore stages. (b) qRT–PCR analysis of *ZmMs7* in wild type (WT) during different anther developmental stages. (c) qRT–PCR analysis of *ZmMs7* in fertile (*Ms7/ms7‐6007*) and sterile (*ms7‐6007/ms7‐6007*) plants during the early‐vacuolate microspore stage. *ZmMs7* expression data were normalized to *ZmActin1*. Different developmental stages of anther are shown: IMT, immature tassels; Q, quartets; EV, early‐vacuolate microspore; MV, middle vacuolate microspore; LV, late vacuolate microspore; H, heading. L, leaves; E, ears; R, roots.

To analyse the expression pattern of *ZmMs7*, semi‐quantitative RT–PCR and quantitative real‐time PCR (qRT–PCR) were performed. The obtained results revealed that *ZmMs7* was specifically expressed in postmeiotic anthers from the quartet to late vacuolate microspore stages, with the highest expression levels in the early‐vacuolate microspore stage (Figure 5a and b). In contrast, *ZmMs7* expression was not detected in anthers at other developmental stages or in the leaf, ear or root during any developmental stages (Figure 5a and b). Compared with the expression of *ZmMs7* in *Ms7/ms7‐6007* male‐fertile siblings, the *ms7‐6007* male‐sterile mutant exhibited lower *ZmMs7* expression at approximately one‐third the expression level (Figure 5c). Together, these results confirmed that *ZmMs7* is an anther‐specific expression gene and may play a specific role during the development of anthers and microspores.

### ZmMs7 is the ortholog of rice PTC1 and *Arabidopsis* MS1

**Figure 6:**
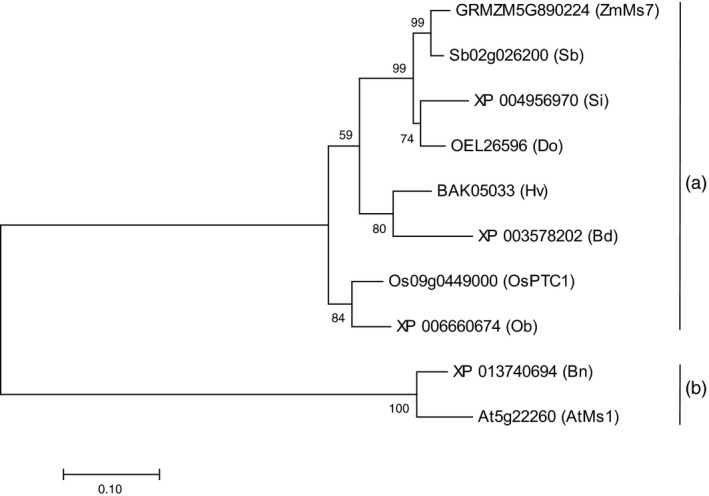
Phylogenetic analysis of ZmMs7 and its related proteins. The evolutionary analyses were conducted in MEGA7 using the maximum‐likelihood method based on the Poisson correction model. The tree with the greatest log likelihood is shown. The analysis involved 10 amino acid sequences from *Zea mays* (Zm), *Sorghum bicolor* (Sb), *Oryza sativa* (Os), *Oryza brachyantha* (Ob), *Arabidopsis thaliana* (At), *Dichanthelium oligosanthes* (Do), *Setaria italica* (Si), *Brachypodium distachyon* (Bd), *Hordeum vulgare* (Hv) and *Brassica napus* (Bn). Group A comprises eight proteins from monocots, and group B includes two proteins from dicots.

The full‐length genomic DNA and cDNA of *ZmMs7* were cloned and sequenced from the B73 maize inbred line (GenBank accession numbers KY657532 and KY657533). The ZmMs7 protein was predicted to comprise 670 amino acids (73.5 kDa). To gain insight into the phylogenetic relationship between ZmMs7 and its close homologs, the ZmMs7 protein sequence was used in BLASTp queries in NCBI. A phylogenetic tree of ZmMs7 and its 9 homologs was constructed (Figure 6). The phylogenetic analysis indicated that maize ZmMs7 was located in the same clade with rice PTC1 (Li *et al*., 2011) and other monocot homologs, such as *Sorghum bicolor*,* Hordeum vulgare*,* Oryza brachyantha*,* Dichanthelium oligosanthes*,* Setaria italica* and *Brachypodium distachyon* (Figure 6a), whereas the *Arabidopsis thaliana* MS1 and *Brassica napus* homolog occupied a relatively distant dicot branch (Figure 6b).

**Figure 7:**
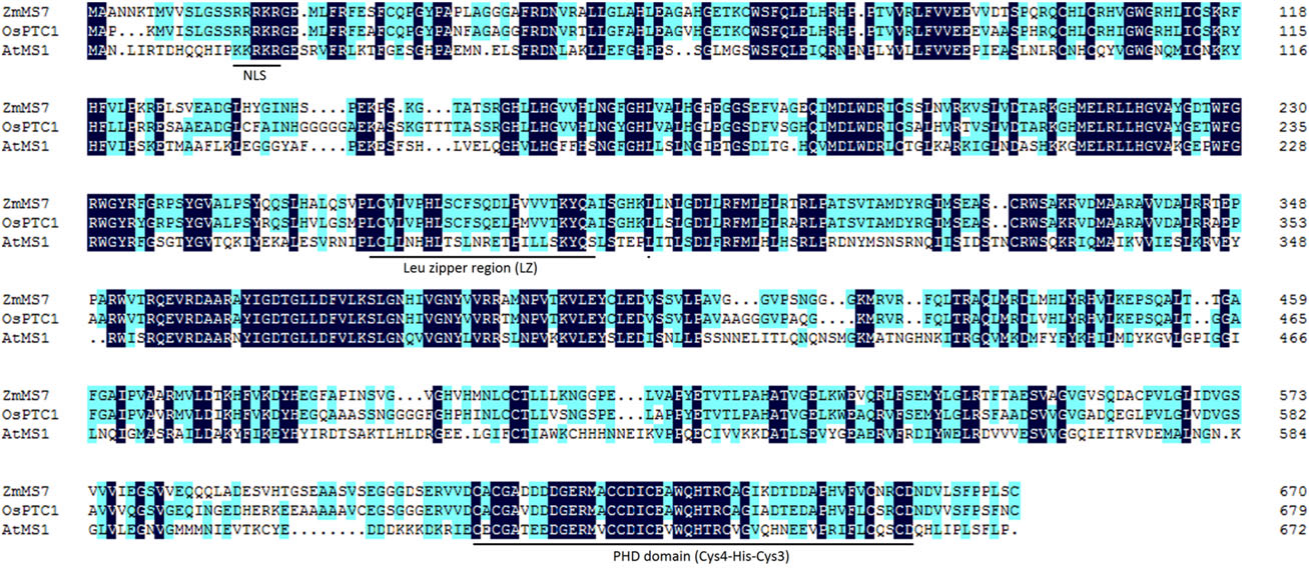
The amino acid sequence alignment of maize ZmMs7, rice PTC1 (OsPTC1) and *Arabidopsis* MS1 (AtMS1). The nuclear localization sequence (NLS), leucine zipper region (LZ) and putative conserved PHD domain (Cys4‐His‐Cys3) are underlined (refer to Li *et al*., 2011; Wilson *et al*., 2001).

Based on the predicted amino acid sequence alignment, ZmMs7 shared 80.85% and 40.49% similarity with rice PTC1 and *Arabidopsis* MS1, respectively. Using the conserved domain BLAST in NCBI, the 624‐658‐amino acid interval of ZmMs7 was predicted as a plant homeodomain (PHD) domain (Figure 7) and was conserved among maize, rice and *Arabidopsis*. Furthermore, based on *Arabidopsis* MS1 (Wilson *et al*., 2001), a nuclear localization signal (RRRKR) was found at the N‐terminus, and the 260‐282‐amino acid interval of ZmMs7 was predicted to be a leucine zipper region (Figure 7), which is required for protein function (Ito *et al*., 2007). Interestingly, the region harbouring the *PTC1* gene on rice chromosome 9 was syntenic to the maize chromosome 7 region containing *ZmMs7* (Li *et al*., 2011). Considered together with the phylogenetic analysis (Figure 6), we concluded that *ZmMs7* encodes a PHD‐finger transcription factor that is orthologous to rice PTC1 and *Arabidopsis* MS1.

### Development of a multicontrol sterility system using *ZmMs7*

As the *ms7‐6007* mutant exhibits complete male sterility and no pollen exsertion from its tassel (Figure 1; Beadle, 1932), it is desirable for use in the development of a male‐sterile line for hybrid seed production. Here, we developed a MCS system based on the *ms7‐6007* mutant and *ZmMs7* gene. The technical strategy is shown in Figure S3a.

First, three p*MCS* binary vectors (p*MCS0701*,* 0702* and *0703*) were constructed (Table 1 and Figure S3b). The T‐DNA region contained four or five functional modules: (i) *ZmMs7* driven by its native promoter for restoration of male fertility, (ii) maize α‐amylase gene *ZmAA* (Wu *et al*., 2016) driven by the pollen‐specific *PG47* promoter (Allen and Lonsdale, 1993) and/or (iii) the DNA adenine methylase gene *Dam* (Brooks *et al*., 1983; Unger *et al*., 2001) driven by the pollen‐specific *Zm13* promoter (Hanson *et al*., 1989) to devitalize the transgenic pollen, (iv) red fluorescence protein gene *DsRed2* or *mCherry* (Matz *et al*., 1999) driven by the aleurone‐specific *LTP2* promoter (Kalla *et al*., 1994) to mark the transgenic seed and (v) the herbicide‐resistant gene *Bar* driven by the *CaMV35S* promoter for genetic transformation and transgenic seed selection.

**Table 1:** Constructs for development of MCS maintainer lines using the *ZmMs7* gene

Construct	Promoter‐gene combination
pMCS0701	*p35S::Bar//pZmMs7::ZmMs7//pZm13::Dam//pPG47::Bt1:ZmAA//pLTP2::mCherry*
pMCS0702	*p35S::Bar//pPG47::Bt1:ZmAA//pZmMs7::ZmMs7//pLTP2::DsRed2*
pMCS0703	*p35S::Bar//pPG47::Bt1:ZmAA//pZm13::Dam//pZmMs7::ZmMs7//pLTP2::DsRed2*

*ZmMs7*, maize fertility restoration gene; *ZmAA*, α‐amylase gene; *DsRed2* and *mCherry*, red fluorescence gene; 35S, cauliflower mosaic virus 35S promoter; *Bar*, herbicide resistance gene; Bt1, Brittle‐1 transit peptide; pZm13, *Zm13* gene promoter; *Dam*, DNA adenine methylase gene; pLTP2, lipid transfer protein‐2 gene promoter; pZmMs7, *ZmMs7* gene promoter; pPG47, polygalacturonase gene promoter.

**Figure 8:**
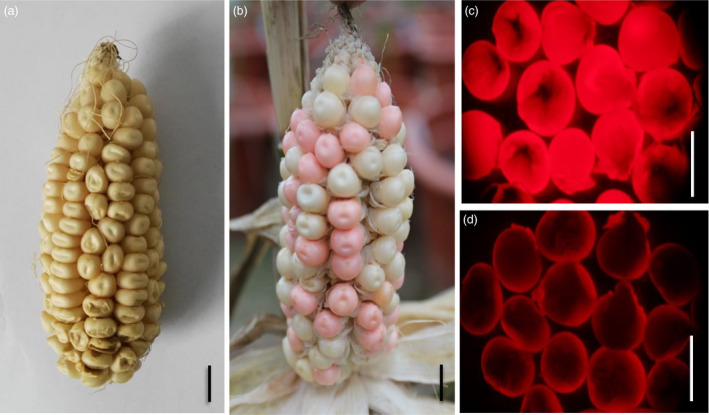
Phenotype of the *ZmMs7* transgenic maintainer line (T_1_). (a and b), Ear phenotype of the maize Hi‐II hybrid line (a) and transgenic event (M0701‐2, b); (c and d), transgenic seeds (c) and nontransgenic seeds (d) from M0701‐2 under a red fluorescence (RF) filter. T_1_ is the first transformation generation. Bars = 1 cm.

Second, the p*MCS* constructs were introduced into the maize Hi‐II hybrid line via *Agrobacterium tumefaciens‐*mediated transformation to obtain *ZmMs7* transgenic intermediate material. The T_0_ transgenic plants were pollinated using the pollen of heterozygous *Ms7/ms7‐6007*, and the T_1_ progenies were screened for events carrying both a single copy of the p*MCS* T‐DNA and the *ms7‐6007* allele. Based on the phenotypic characterization, RT–PCR and Southern blotting analysis (Figure S4), an elite T_1_ transgenic event of p*MCS0701* (M0701‐2) was selected as a representative and used for further study. The M0701‐2 plant exhibited normal vegetative and reproductive growth, and a nearly 1:1 (59:61) ratio of fluorescent seeds (with a hemizygous transgene) to nonfluorescent seeds (no transgene) on the ear (Figure 8). The seeds were sorted out manually based on red fluorescence and grown to obtain the next generation.

Third, the transgenic event M0701‐2 was self‐pollinated and screened for the homozygous genotype of the *ms7‐6007* allele, and then, a candidate male‐sterile maintainer line for *ms7‐6007* was obtained. Because *ZmMs7* is a sporophytic male fertility gene, a hemizygous *ZmMs7* transgene in *ms7‐6007* can fully restore male fertility. As *ZmAA* driven by a *PG47* promoter is a gametophytic factor that disrupts starch accumulation only in the transgenic pollen (Allen and Lonsdale, 1993; Wu *et al*., 2016), and anther‐targeted expression of *Dam* in maize is used to inactivate a genetic region critical for pollen formation or function thereby causing abnormal development of tapetal cells and microspores (Cigan and Albertsen, 2001; Unger *et al*., 2001), all the transgenic pollen grains produced by the hemizygous transgenic plants were deactivated and infertile. The selected T_2_ plant was homozygous at the *ms7‐6007* locus but hemizygous at the MCS T‐DNA locus. As pollen grains carrying the transgenic T‐DNA were all defective, the transgenic element was inherited only through the female gamete. All plants from the fluorescent seeds were male fertile, but all plants from the nonfluorescent seeds were male sterile. The fluorescence screening and fertility examination experiments were repeated for three generations, with a total of more than 500 000 seeds, all of which showed the same results as described above; namely, all fluorescent seed‐derived plants were male fertile, but all nonfluorescent seed‐derived plants were male sterile, indicating that both the transgenic p*MCS0701* and *ms7‐6007* mutant locus elements were inherited stably.

### Confirmation of whether MCS progeny seeds inherit the transgene through pollen

To determine the transgene transmission risk through pollen, transgenic MCS maintainer plants from three constructs (differing by the number of pollen‐disrupted genes, the gene orientation and the seed colour marker gene; Table 1) were used to pollinate the maize inbred line Zheng58, which is the female parent line of Zhengdan 958, a famous maize hybrid with the largest planting area in the last decade in China. Ears were harvested from the female parent plants and examined under green excitation light for the presence of red fluorescent seeds resulting from the expression of DsRed2 or mCherry protein. The transgene transmission rate through pollen in seeds varied with different constructs and different transformant events (Table 2). The percentages of red fluorescent seeds in the four events of pMCS0701 were varied from 0.031% to 0.037% (averagely 0.033%), those in five events of pMCS0703 were varied from 0.032% to 0.043% (averagely 0.036%), while those in three events of pMCS0702 were varied from 0.236% to 0.301% (averagely 0.263%). In summary, the constructs of p*MCS0701* and p*MCS0703*, containing two pollen‐disrupted modules (*ZmAA* and *Dam*), showed significantly lower rates of transgenic pollen transmission compared with the construct of p*MCS0702* harbouring one pollen‐disrupted module (*ZmAA*). This result suggests that two pollen‐disrupted modules are more efficient than one pollen‐disrupted module in preventing transgene transmission through pollen in the genetic background of maize inbred line Zheng58. Subsequently, transformant M0701‐2 from the p*MCS0701* construct (Table 1) was selected as the MCS maintainer line to produce the maize male‐sterile lines, which are very useful in maize breeding and hybrid seed production.

**Table 2:** Transgene transmission rate through the pollen of different *ZmMs7* transgenic maintainer lines

Construct	Generation	Transformants	Number of red fluorescent seeds/total number of seeds produced by nontransgenic plants pollinated by transgenic plants	Per cent of red fluorescent seeds (%)
pMCS0701	T_2_	M0701‐2	58/157 552	0.037
		M0701‐9	26/83 871	0.031
		M0701‐25	14/41 176	0.034
		M0701‐37	11/34 874	0.0315
pMCS0702	T_2_	M0702‐3	224/94 751	0.236
		M0702‐6	78/31 076	0.251
		M0702‐11	53/17 608	0.301
pMCS0703	T_2_	M0703‐6	204/578 682	0.035
		M0703‐9	31/96 875	0.032
		M0703‐16	28/74 684	0.038
		M0703‐22	19/43 901	0.043
		M0703‐23	14/4 0967	0.034

T_2_ is the second‐generation transformant. There are two pollen‐disrupted genes (*ZmAA* and *Dam*) in the pMCS0701 and pMCS0703 constructs but only one pollen‐disrupted gene (*ZmAA*) in the pMCS0702 construct (refer to Table 1).

### Application of the M0701‐2 maintainer line for breeding various male‐sterile lines in maize

To breed various male‐sterile lines by transferring the *ms7‐6007* mutant locus and the MCS transgenic T‐DNA element into other germplasm, M0701‐2 (as the maternal parent) was crossed with five different maize elite inbred lines (including Zheng58) as the recurrent male parent, respectively. Progeny were analysed for the presence of the *ms7‐6007* mutant locus and transgenic element by marker‐assisted selection, and seeds of two consecutive backcrossed and self‐pollinated generations (BC_2_F_2_) have been obtained to date. Both the *ms7‐6007* mutant locus and the transgenic element were accurately associated with their respective phenotypes in the segregated progeny. The ears of three candidate MCS transgenic maintainer lines are shown in Figure S5. The ratio of red fluorescent seeds to nonfluorescent seeds in each ear was nearly 1:1, while all red fluorescent seeds were male fertile, and nonfluorescent seeds were male sterile. These results indicated that both the *ms7‐6007* mutant locus and the transgenic element from the M0701‐2 event maintained their respective functions when transferred into different genetic backgrounds in maize, making it feasible to breed male‐sterile lines using traditional backcrossing and marker‐assisted selection methods.

## Discussion

### The *ms7* mutant displays complete recessive male sterility, an abnormal microspore wall and tapetal cell development

*ms7* is a complete recessive male‐sterile mutant with no exserted anthers (Figure 1b, c, d; Table S1) and has been characterized cytologically as having a poorly developed, thin microspore wall and aperture as well as abnormal tapetal cell development (Morton *et al*., 1989). The tapetal cells in the *ms7* mutant did not become flattened tangentially to the anther wall layers as in normal tapetal cell development, and darkly staining material was observed within the *ms7* mutant cells. Microsporogenesis was normal until the quartet stage when precocious chromosome condensation might occur (Albertsen and Phillips, 1981). Similarly, tapetal cells of the *ms7* mutant appeared normal up to the quartet stage when they started to vacuolate and rapidly degenerate (Morton *et al*., 1989). Consistent with previous reports, our data showed that during the late microspore developmental stage, the tapetal cells of the *ms7‐6007* mutant were swollen and lightly stained with aborted microspores, leaving debris in the shrinkage locule and indicating abnormal PCD of tapetal cells (Figure 1e, f). Furthermore, the cytological characteristics of the *ms7* mutant were similar to the rice *ptc1* and *Arabidopsis ms1* mutants, which displayed delayed tapetum degeneration and defects in the development of the pollen exine (Ito and Shinozaki, 2002; Ito *et al*., 2007; Li *et al*., 2011; Yang *et al*., 2007). Together, these results suggested that *ZmMs7* played important roles in microspore wall formation and tapetal cell PCD during late anther development. However, until now, the *ZmMs7* gene has not been cloned or characterized.

### *ZmMs7* encodes a PHD‐finger protein similar to rice PTC1 and *Arabidopsis* MS1

Using a map‐based cloning approach, we found that *ZmMs7* encodes a PHD‐finger protein with a N‐terminal nuclear location signal and a leucine zipper‐like region, which shares high similarity with rice PTC1 and *Arabidopsis* MS1 (Figure 7). All three mutants (maize *ms7*, rice *ptc1* and *Arabidopsis ms1*) showed similar male‐sterile phenotypes, defects in pollen exine formation and tapetal cell development (Figure 1) (Ito and Shinozaki, 2002; Ito *et al*., 2007; Li *et al*., 2011; Morton *et al*., 1989; Yang *et al*., 2007). Moreover, similar to the spatio‐temporal expression patterns of rice *PTC1* and *Arabidopsis MS1* genes, *ZmMs7* was also expressed specifically in maize anthers from the quartet to the late vacuolate microspore stages (Figure 5). Furthermore, the orthologs of ZmMs7 were found in nine flowering plants by a BLASTp search and phylogenetic analysis (Figure 6). Based on the cytological similarity of mutants, the same expression pattern of genes and the high identity of the proteins, we conclude that *ZmMs7* gene is the ortholog of the rice *PTC1* and *Arabidopsis MS1* genes, and they are functionally conserved in flowering plants. Mutations in the three genes (maize *ZmMs7*, rice *PTC1* and *Arabidopsis MS1*) led to a similar male‐sterile phenotype and cytological defect, demonstrating that this kind of PHD‐finger protein is required for plant male gametogenesis and has potential application value in crop hybrid seed production.

### The technical strategy of the MCS system based on the *ZmMs7* gene

To date, hundreds of recessive genetic male‐sterile mutants have been identified in maize, but their application in maize breeding and hybrid seed production has been limited because of the inability to propagate a pure male‐sterile line via self‐pollination. As more male‐sterile genes have been cloned and an efficient transgenic method becomes feasible in maize, several strategies for maintaining and propagating male‐sterile lines have been proposed based on the transgenic maintainer line (Perez‐Prat and van Lookeren Campagne, 2002; Williams, 1995; Wu *et al*., 2016). Among them, the Seed Production Technology (SPT) strategy devised by DuPont‐Pioneer has been used for commercial hybrid seed production (Wu *et al*., 2016). The core technology of SPT system is the transformation of the male‐sterile mutant *ms45* with a SPT construct, which comprises three functional modules: (i) a male fertility gene, (ii) a pollen‐disrupted gene and (iii) a seed colour marker gene. Although many advantages of the SPT system have been suggested, such as no need detasseling, no limitations to its use in maize germplasm and an increased hybrid seed purity, and even enhanced hybrid seed production yields, the transgene transmission rate through pollen (based on red florescence seeds expressing the *DsRed2* gene) has been observed to vary with different SPT constructs, transformant events and genetic backgrounds, with the range of transgene transmission rates from 0.002% to 0.518% (Wu *et al*., 2016).

To decrease the transgene transmission rate of the transgenic maintainer line through pollen, we developed a MCS system by transforming MCS constructs into the *ms7‐6007* mutant (Figure S3). The MCS constructs contained (i) the cloned male fertility gene *ZmMs7* to restore fertility, (ii) two pollen‐disrupted genes (*ZmAA* and *Dam*) to disrupt the transgenic pollens, (iii) the screenable fluorescent colour marker gene (*DsRed2* or *mCherry*) to identify the transgenic seeds and facilitate purification of the transgenic MCS maintainer line and (iv) the herbicide‐resistant gene (*Bar*) to prevent sophistication of MCS maintainer line seeds (Figure S3). Because the MCS construct harbours two pollen‐disrupted modules, both of which can inhibit transgenic pollen formation or function, the transgene transmission rate through pollen can be greatly decreased. One is the *ZmAA* gene driven by the late pollen‐specific *PG47* promoter (Allen and Lonsdale, 1993; Wu *et al*., 2016), which can interfere with normal starch accumulation in transgenic pollen and repress pollen development. The other is the *Dam* gene driven by the pollen‐specific *Zm13* promoter (Brooks *et al*., 1983; Hamilton *et al*., 1992; Hanson *et al*., 1989; Unger *et al*., 2001), which catalyses the methylation of adenine residues in the DNA of pollen and affects the cell viability of transgenic pollen. Anther‐targeted expression of *Dam* gene results in abnormal tapetal cells and microspores and renders maize male sterile (Unger *et al*., 2001). Our data indicated that the transgene transmission rate through pollen in MCS transformants harbouring two pollen‐lethality genes (*ZmAA* and *Dam*) decreased significantly (1/8–1/7) compared with those harbouring one gene (*ZmAA*) in the genetic background of Zheng58 (Table 2), confirming that the MCS constructs containing two pollen‐lethality genes were more efficient and viable in the genetic background of Zheng58. Furthermore, there are several advantages of *Bar* in the MCS construct to propagate highly pure seeds of the MCS transgenic maintainer line and male‐sterile line. During the MCS transgenic maintainer line‐propagating phase, to assure the high purity (100%) of the MCS maintainer line seeds, appropriate herbicide spraying of seedlings of the MCS maintainer line in the field will efficiently eliminate any nontransgenic plants. During the male‐sterile line‐propagating phase, the male‐sterile line is cross‐pollinated with the transgenic maintainer line as pollen donor. Additionally, it is useful to spray herbicide specifically on the transgenic maintainer line to eliminate the mixed nontransgenic plants, which ensures a high purity of male parent pollen and male‐sterile line seeds. Compared with the SPT, the MCS constructs harbouring the five functional modules contain a *Bar* and a *Dam* gene, which can assure high purity of the male‐sterile parent line and hybrid seed production, and greatly decrease the transgene transmission rate as well as the transgene flowing risk. Therefore, this strategy was designed as a multicontrol sterility system.

The MCS system is superior to CMS and other biotechnology‐based systems in several aspects. First, compared to the CMS system, the MCS male‐sterile line is controlled by a single recessive nuclear gene, and any maize germplasm carrying the wild‐type *ZmMs7* allele can complement the *ms7‐6007* mutation. Moreover, the male sterility is genetically stable in various environments, greatly reducing the risk induced by weather changes. Second, compared with the SPT system, the transgenic MCS maintainer lines contain five functional modules, in particular, two pollen‐disrupted genes (*ZmAA* and *Dam*) driven by two pollen‐specific promoters, *PG47* and *Zm13*, respectively, which significantly reduce the transgene transmission rate through pollen (Table 2). Third, the herbicide resistance gene *Bar* in the MCS system makes it easier to transfer and select transgenic constructs in maize germplasm with different genetic backgrounds by appropriate herbicide spraying of the seedlings to eliminate nontransgenic plants. Concurrently, herbicide resistance in the MCS system is beneficial for the propagation of high‐purity MCS transgenic maintainer line seeds and male‐sterile line seeds through herbicide spraying during specific stages of production. Finally, although the MCS system involves transgenic processes, only the MCS maintainer line carries the transgene. Thus, neither the obtained male‐sterile line seeds nor the hybrid seeds contain any transgenic elements.

Acknowledgements of the nontransgenic status of progeny produced by the SPT process have been supported by regulatory agencies in the USA (USDA‐APHIS, 2011), Australia (FSANZ, 2012) and Japan (MHLW, 2013). Compared with the SPT system, the transgene transmission rate through the MCS maintainer pollen may be reduced in a specific genetic background, and the herbicide resistance in MCS maintainer lines makes them safer and more efficient for the production of high‐purity male‐sterile parent lines and hybrid seeds. Therefore, the MCS system should also be considered for a nontransgenic status to facilitate the use of MCS male‐sterile lines in commercial maize hybrid seed production, especially in China. First, there are more than 200 000 hectares of maize hybrid seed production acreage every year in China, and commercial application of the MCS system will dramatically reduce the total labour costs. Second, hybrid seed production yields can be increased significantly due to the absence of a need for physical (manual and/or mechanical) detasseling, which often removes the tassel together with several leaves, resulting in reductions by as much as 40% of the potential seed yield (Wych, 1988). Third, based on the MCS male‐sterile line, the efficiency of hybrid maize breeding will be greatly accelerated by the increased level of hybrid combination per breeder through the use of the open‐pollination approach in different isolation fields. Finally, considering the huge planted acreage (approximately 34 million hectares) of maize in China, the total yield of commodity maize grain using MCS hybrid seeds will be significantly increased because of the high quality and purity of hybrid seeds produced from the MCS system. Therefore, commercial application of the MCS technology will greatly enhance the efficiency of maize hybrid breeding, increase hybrid seed yields and even raise maize grain commodity yields.

### Potential applications of the MCS system in other major crops

Based on BLASTp and phylogenetic analyses, several function‐conserved orthologs of ZmMs7 have been found in other major crops, including sorghum, barley and oilseed rape (Figure 6), which have flowers that are not amenable to manual emasculation. Therefore, a mutation generated artificially in orthologous genes (*Sb02 g026200*,* BAK05033* and *XP_013740694*, Figure 6) may also cause male sterility. Recently, *MALE STERILITY1* (*HvMS1*), the ortholog of *Arabidopsis MS1* and rice *PTC1,* has been isolated using RACE‐PCR and subsequent functional testing using RNAi lines in barley (Fernández Gómez and Wilson, 2014). Thus, a reverse genetic approach could be adopted using targeted mutagenesis technologies such as the programmable DNA endonuclease and CRISPR/Cas9 system (Cigan *et al*., 2017; Gomez *et al*., 2015; Svitashev *et al*., 2016), which will lead to site‐specific mutations in the corresponding orthologs of ZmMs7 and produce male‐sterile mutants in these crops. The MCS system could be transferred into other major crops using artificial *ms* mutants and the corresponding fertility restoration genes. In summary, the MCS system will greatly expand the potential to produce male‐sterile lines and hybrid seeds of important crops.

## Experimental procedures

### Plant materials and growth conditions

*ms7‐6007* and *ms7gl1* mutants were obtained from the Maize Genetics Cooperation Stock Center. The two F_2_ mapping populations were derived from crosses of *ms7‐6007 *×* *Chang7‐2 and *ms7gl1 *×* *Chang7‐2, respectively. All the plants were grown in the field in Beijing or Sanya, China. The T_0_ transgenic plants and their progeny were grown in a greenhouse in Beijing, China.

### Phenotypic identification, histochemical analysis and microscopy

Tassels and spikelets were photographed using a Canon EOS 700D digital camera (Canon, Japan). To evaluate the pollen viability, anthers and pollen grains were stained with 1% I_2_‐KI and photographed under an Olympus SZ51 microscope (Olympus, Japan). For transverse section analysis, the spikelet was fixed in 3:1 ethanol: acetic acid. Fixed samples were dehydrated through an ethanol series and embedded in epoxy resin. Semi‐thin sections were obtained using an Ultracut E Ultramicrotome (Reichert), stained with 0.1% toluidine blue O and observed under an Olympus BX61 microscope (Olympus, Japan).

### Map‐based cloning

Genomic DNA was extracted from maize leaves using the CTAB method with some modifications (Murray and Thompson, 1980). Nine SSR primers on maize chromosome 7L were chosen for *ms7* primary mapping (Table S2). For fine mapping, several CAPS markers were designed with DNAMAN6.0 (LynnonBiosoft) (Table S3). All PCR primers for these SSR and CAPS markers were synthesized by Sangon Biotech (Shanghai, China) and were tested to identify polymorphic markers distinguishing fertile from sterile plants. By scoring the presence/absence of recombinants at diverse marker locations, the *ms7* gene was narrowed to a 180‐kb interval on chromosome 7L.

### Protein alignment and phylogenetic analysis

Ten homologs of ZmMs7 were obtained by BLASTp search at the NCBI website. The phylogenetic tree was generated in MEGA7.0 (Kumar *et al*., 2016) using the maximum‐likelihood method with the following parameters: Poisson model, complete deletion and 1000 bootstrap replications. The amino acid sequences of maize ZmMs7, rice PTC1 and *Arabidopsis* MS1 were aligned using DNAMAN6.0, and the conserved domains were analysed with a CD search in the NCBI website.

### RNA extraction and expression analysis

Total maize RNA was isolated using TRIzol reagent (Invitrogen) from the following maize tissues: anthers during different stages, leaves, roots and immature ears. Total RNA (1 μg) was used to synthesize first‐strand cDNA using Superscript III RT (Invitrogen). RT–PCR analyses were conducted using 1 μL cDNA as template. qRT–PCR analyses were performed using SYBR Green PCR Master Mix (ABI). *ZmActin1* was used as the internal control. The relative expression was calculated using the 2^−▵▵Ct^ method. The SD was calculated with three biological replications. All primers used for RT–PCR and qRT–PCR are listed in Table S4.

### Plasmid construction, transformation and transgene determination

For complementation, the *ZmMs7* gene promoter (1.2 kb) was amplified from maize B73 using primer Ms7‐ProP, and the *ZmMs7* coding sequence (2 kb) was amplified using primer Ms7‐CDSP from the cDNA of B73 anthers. The two fragments were fused together with *Hin*dIII/*Bam*HI and then cloned into *pBCXUN* (Chen *et al*., 2009). The vector was named *pZmMs7pro::ZmMs7*.

The T‐DNA of the MCS constructs was inserted into the backbone of *pCAMBIA3301* (www.cambia.org) and named *pMCS0701*,* pMCS0702* and *pMCS0703* respectively. The detailed procedure refers to Data S1.

All constructs were transformed into the maize Hi‐II hybrid line using the *Agrobacterium‐*mediated transformation method (Frame *et al*., 2002). The *Bar* gene was employed as a selectable marker, and the transformants were screened by PCR amplification using the primer Bar‐P. The transgenic T‐DNA region was then transferred into the *ms7‐6007* mutant by the crossing and backcrossing method. To confirm the presence of the *ms7‐6007* allele and transgenic T‐DNA sequence in the progeny, the transgenic plants were screened by PCR amplification using primers ms6007‐ID and Bar‐P. The genomic DNA of each transgenic plant was digested with *Hind*III and further analysed using a DIG High Prime DNA labelling and Detection Starter Kit II (Roche) with DIG‐labelled *mCherry* as the probe for Southern blotting analysis. Expression of the *mCherry* gene was determined by RT–PCR using mCherry‐P primer, and *ZmActin1* was used as the native control. All the above‐described primers are listed in Table S4.

### Seed colour sorting

A dual‐fluorescent protein (DFP) flashlight (www.nightsea.com/products/dfp) was used for seed colour sorting. With the matching barrier filter glasses, the red fluorescent seeds could be easily sorted from the nonred fluorescent seeds. The number of red fluorescent seeds in different transformants was counted manually.

## Supporting information


**Figure S1** Phenotypic comparison of the wild type (WT) and *ms7gl1* mutant.
**Figure S2** Predicted amino acid sequences and alignment of *ZmMs7* in wild type and the *ms‐6007* and *ms7gl1* mutants.
**Figure S3** A multi‐control sterility (MCS) system in maize via a transgenic approach.
**Figure S4** Molecular analysis of *ZmMs7* transgenic maintainer lines.
**Figure S5** Phenotype of three *ZmMs7* transgenic maize male‐sterile maintainer lines (BC_2_F_1_).
**Table S1** The ratio of fertile to sterile plants in the F_2_ population of the *ms7* mutant.
**Table S2** The SSR marker information used for *ZmMs7* primary mapping.
**Table S3** The CAPS marker information used for *ZmMs7* fine mapping.
**Table S4** PCR primers used in this study.
**Data S1** The detailed construction procedure used for the three MCS plasmids.
