# Supplementary material for: Construction of a multicontrol sterility system for a maize male‐sterile line and hybrid seed production based on the ZmMs7 gene encoding a PHD‐finger transcription factor
Source: Plant Biotechnol J. 2017 Aug 23;16(2):459–71. doi: 10.1111/pbi.12786 (PMC5787847; doi:10.1111/pbi.12786)
Supplement: Supplementary file 1 — Figure S1 Phenotypic comparison of the wild type (WT) and ms7gl1 mutant. Figure S2 Predicted amino acid sequences and alignment of ZmMs7 in wild type and the ms‐6007 and ms7gl1 mutants. Figure S3 A multi‐control sterility (MCS) system in maize via a transgenic approach. Figure S4 Molecular analysis of ZmMs7 transgenic maintainer lines. Figure S5 Phenotype of three ZmMs7 transgenic maize male‐sterile maintainer lines (BC2F1). Table S1 The ratio of fertile to sterile plants in the F2 population of the ms7 mutant. Table S2 The SSR marker information used for ZmMs7 primary mapping. Table S3 The CAPS marker information used for ZmMs7 fine mapping. Table S4 PCR primers used in this study. Data S1 The detailed construction procedure used for the three MCS plasmids. [file PBI-16-459-s001.doc]

**Construction of a multi-control sterility system for a maize male-sterile line and hybrid seed production based on the *ZmMs7* gene encoding a PHD-finger transcription factor**

**Supporting Information**

**Figure S1.** Phenotypic comparison of the wild type (WT) and *ms7gl1* mutant. (a and b) The tassel of WT (a) and *ms7gl1* (b). (c) The spikelet of WT (left) and *ms7gl1* (right) with the glume, lemma and palea removed. (d) The anther of WT (left) and *ms7gl1* (right) stained with I_2_-KI. Bars=1 mm.

**Figure S2.** Predicted amino acid sequences and alignment of *ZmMs7* in wild type and the *ms-6007* and *ms7gl1* mutants. The insertion mutations in *ms7-6007* and *ms7gl1* caused frame-shift mutations and an altered reading frame after amino acids 157 and 261, respectively, resulting in the absence of the conserved leucine zipper region (LZ) and PHD domains.

**Figure S3**. A multi-control sterility (MCS) system in maize via a transgenic approach. (a) Schematic representation of the MCS system based on the *ms7* mutant line and MCS constructs. (b) The p*MCS* plasmids (p*MCS0701*, p*MCS0702*, p*MCS0703*) for transformation. The T-DNA of MCS constructs containing four (p*MCS0702*) or five (p*MCS0701* and p*MCS0703*) functional modules, including (i) the wild-type fertile gene (*ZmMs7*) to restore male fertility, (ii) the α-amylase gene (*ZmAA*) and/or DNA adenine methylase gene (*Dam*) to devitalize transgenic pollens, (iii) the red fluorescence protein gene (*DsRed2 or mCherry*) and (iv) the *Bar* gene as a seed-screenable marker gene.

**Figure S4.** Molecular analysis of *ZmMs7* transgenic maintainer lines. (a) RT-PCR analysis of three maintainer lines. *mCherry* was used as the target gene, and *ZmActin1* was used as the native control. (b) Southern blotting of three maintainer lines. DIG-labeled *mCherry* DNA was used as the hybridization probe. 1-3, represents the M0701-2, M0701-9, M0701-25 transgenic maintainer lines, respectively.

**Figure S5.** Phenotype of three *ZmMs7* transgenic maize male-sterile maintainer lines (BC_2_F_1_). (a, c and e) Ear photos of the three male-sterile maintainer lines (1601#, 1602# and 1603#) under bright light (BL). (b, d and f) The ear photos corresponding to (a, c and e) under a red fluorescence (RF) filter, respectively. These lines are the BC_2_F_1_ progeny of the elite line M0701-2 crossed with the *ms7-6007* mutant and then backcrossed to the inbred line Zheng58. BC_2_F_1_ is the second backcrossed generation. Bars=1 cm.

**Table S1.** The ratio of fertile to sterile plants in the F_2_ population of the *ms7* mutant.

| F_2_ population combination | Total plants | Fertile plants(F) | Sterile plants(S) | F/S ratio | χ2 | P | Significant test, P>0.05 |
| --- | --- | --- | --- | --- | --- | --- | --- |
| *ms7gl1*×Chang7-2 | 691 | 525 | 166 | 3.16:1 | 0.1782 | 0.6729 | ns* |
| *ms7-6007*×Chang7-2 | 995 | 763 | 232 | 3.29:1 | 0.6173 | 0.4321 | ns* |

**Table S2.** The SSR marker information used for *ZmMs7* primary mapping.

| **Primers** | **Forward（5’-3’）** | **Reverse（5’-3’）** |
| --- | --- | --- |
| umc1016 | GTGATACCGGGTAATCTGGTGC | GATGATGGGTGATCATCGGTTC |
| umc2572 | CGTCTTCGTCACCGTCTCTTG | CAACCAATCTACTCGCTGCTACAA |
| bnlg1792 | GCGCTCCTTCACCTTCTTTA | GCGCTCCTTCACCTTCTTTA |
| umc2617 | GTGGCTCCTCGTATATTCATCGAC | GAAAAGAAAGAGAGAAAGGGCAGG |
| bnlg1808 | CTTTTCTCTTCTAGTAATGAACAGTCA | GCATGATCGAACGAAGGC |
| mmc0411 | CGATGCAAGAGTGTCAAGTA | ACTCCCTAGTGCAAAAATCA |
| umc1450 | ACTTTATTTAGCCCACGTCACTCG | AGTATGACACGGGATTTTGCTGTT |
| bnlg1305 | GCACGGGCATCAGAGAGAG | CATGGGTAAGTTGCTGAAAGTTT |
| umc2576 | AGGAAGCAGTAGGCGACGTAGAG | CTACGGCTACGGGATCGTTTTC |

**Table S3.** The CAPS marker information used for *ZmMs7* fine mapping.

| Primer | Gene resource | Restriction enzyme | Primer sequence (5’-3’) |
| --- | --- | --- | --- |
| EP222 | GRMZM2G050172 | *Hpa* II | F: TACTCGCACTCCCACTCGTCT |
|  |  |  | R: GCACTCAGATGGAGGTTGGAA |
| EP238 | GRMZM2G154752 | *Hpa* II | F: GAACGGACACGAACACGATC |
|  |  |  | R: GACCTGACATAGTAAGGCCAGTT |
| EP239 | GRMZM5G890224 | *Sau3A* I | F: ATCGCCAATACAATGAACAGC |
|  |  |  | R: TGGATAACCAAACGAAACACG |
| EP259 | GRMZM2G141031 | *Sau3A* I | F: CAACCATTGATTTGGGCTCA |
|  |  |  | R: TGACCAGGGAGACTTTATTGCA |
| EP299 | MAGIv3.1_19968 |  | F: CTGGCACGAAGGCTGGTAA |
|  |  |  | R: CTCTCGCCGGTCACTTGATA |
| EP302 | MAGIv3.1_90113 |  | F: GAAGAGGACTTGAACGAGGGAT |
|  |  |  | R: AACCTCCATAATGAATTTCACCTC |

**Table S4.** PCR primers used in this study.

| **Primer** | **Forward (5'-3')** | **Reverse (5'-3')** | **Purpose** |
| --- | --- | --- | --- |
| Ms7-FL | ATGGCTGCCAATAATAAGACGA | CTCACCTTCCTTGCAATGGATAAC | *ZmMs7* full length gDNA and cDNA |
| ms6007-ID | GGCCACAAGCTGCTCAACCT | CTACCTTCCTTGCAATGGATAAC | Identification of *ms7-6007* |
| Ms7-RT | GGCCACAAGCTGCTCAACCT | ATGTGGTTGCCCAGGGACTT | RT-PCR of *ZmMs7* |
| ACTIN-RT | AAATGACGCAGATTATGTTTGA | GCTCGTAGTGAGGGAGTACC | RT-PCR and qPCR of *ZmACTIN* |
| Ms7-qPCR | GGCCACAAGCTGCTCAACCT | CTACCTTCCTTGCAATGGATAAC | qPCR of *ZmMs7* |
| Ms7-FL2 | TGCTGAACAGATTCGTTTGACTC | CTCACCTTCCTTGCAATGGATAAC | Promoter and full length of *ZmMs7* |
| Bar-P | TCTACCATGAGCCCAGAAC | TCAAATCTCGGTGACGGGCA | Transgene determination |
| mCherry-P | ATGGTGAGCAAGGGCGAGGAG | TACTTGTACAGCTCGTCCATGCC | RT-PCR of mCherry |
| Ms7-ProP | TTCGTTTGACTCGGACAAGTTACGTG | GGCAGCCATCCATGGTCGCCGGACCGGACGCCGCG | Vector, p*ZmMs7pro::ZmMs7* |
| Ms7-CDSP | CGGCGACCATGGATGGCTGCCAATAATAAGACGATG | TCGGATCCCTAACAGCTCAAGGGAGGG | Vector, p*ZmMs7pro::ZmMs7* |
| MCS-P1 | CAAAGCTTCTCTAGAACTAGTGGATCTCGATGTGTAG | CTGGTCACCAGATCTTACTCGGCTACACTCACAC | Vector, pMCS0701 |
| MCS-P2 | CGGAGATCTATGGTGAGCAAGGGCGAGGAG | CGGAGATCTTACTTGTACAGCTCGTCCATG | Vector, pMCS0701 |
| MCS-P3 | gtccggtccggcgaCCATGGCTGCCAATAATAAGACG | GAGCCCTGGCATGCCCTAACAGCTCAAGGGAGGGAAT | Vector, pMCS0701 |
| MCS-P4 | ccttgagctgttagGGCATGCCAGGGCTCTCAATGGAG | CCCGGGTCAATCAGTAAATTGAACGGAG | Vector, pMCS0701 |
| MCS-P5 | CCGCCTAGGTTCGTTTGACTCGGACAAGTTACG | TTGGCAGCCATGGTCGCCGGACCGGACGCCGC | Vector, pMCS0701 |
| MCS-P6 | GCTGCCATTTAATGATTCTATATATACTATTC | AAGCGGCCGCCTAGGCCGCATTCGCAAAACACACC | Vector, pMCS0701 |
| MCS-P7 | CGAAGCTTCCCTAGGAGGCGCGCCGGATCTCGATGTGTAGTCTACGAG | AGCTGGTCACCCTACAGGAACAGGTGGT |  |

**Data S1.** The detailed construction procedure used for the three MCS plasmids.

To develop the multi-control sterility system, three binary vectors were constructed: p*MCS0701*, p*MCS0702* and p*MCS0703*. To construct the binary vector p*MCS0701*, the *LTP2* promoter was amplified from barley using primers MCS-P1 and cloned into p*CAMBIA3301* between *Hin*dⅢ and *Bst*EⅡ, resulting in the p*CLTP2* construct. The *mCherry* fragment was amplified with primers MCS-P2 and cloned into *pCLTP2* cut with *Bgl*Ⅱ to generate p*CLC*. The fragment containing the *PG47* promoter, α-amylase gene *ZmAA* and the *IN2-1* terminator was synthesized and then cloned into p*CLC* between *Hin*dⅢ and *Eco*RΙ to generate p*CLCAA*. The *ZmMs7* CDS fragment was amplified with primers MCS-P3 using cDNA from maize B73 anthers, the *OCS* terminator fragment was amplified with primers MCS-P4, and the promoter of *ZmMs7* was amplified from maize using the MCS-P5 primer set. The fragments were fused together and cloned into *pEASY-T5* to generate the plasmid of p*TMs7Ocs*. The *Dam* functional module containing the *Zm13* promoter, *Dam* gene and *PinII* terminator was synthesized and amplified with the MCS-P6 primer sets and then cloned into p*TMs7Ocs* between *Sma*Ι and *Not*Ι to generate p*TMs7OcsDam*. The p*TMs7OcsDam* was digested with *Avr*Ⅱ and the fragment was cloned into p*CLCAA* at the *Avr*Ⅱsite, forming the vector of p*MCS0701*.

To construct the binary vector of p*MCS0702* and p*MCS0703*, the functional module containing the *PG47* promoter, α-amylase gene *ZmAA* and the *IN*2-1 terminator was synthesized and then cloned into *pCambia3301* between *Hin*dⅢ and *Eco*RI to generate p*CPAI*. The *LTP2-DsRed2* fragment was amplified with primers MCS-P7 and then digested with *Hin*dⅢ and *Bst*EⅡ, and cloned into p*CPAI* between *Hin*dⅢ and *Bst*EⅡ sites to generate p*CPAI-LD*. The *ZmMs7* functional module was obtained from the complementation construct of *pZmMs7pro::ZmMs7* (digested with *Avr*Ⅱ and *Asc*Ι) and was cloned into the p*CPAI-LD* between *Avr*Ⅱ and *Asc*Ι. The resulting plasmid was designated p*MCS0702*. An *Avr*Ⅱ-digested fragment containing the *Zm13pro-Dam-Pin*II cassette from plasmid p*MD18-Dam* was inserted into the *Avr*Ⅱ site of plasmid p*MCS0702*. The resulting plasmid was designated p*MCS0703*.
